# Current status and prospects of diabetes mellitus induced erectile dysfunction: A bibliometric and visualization study

**DOI:** 10.3389/fendo.2023.1168744

**Published:** 2023-03-30

**Authors:** Lei Zhang, Binghao Bao, Jianqiang Guo, Zhongjian Qin, Haonan Huang, Lu Chen, Baoxing Liu

**Affiliations:** ^1^ Graduate School of Beijing University of Chinese Medicine, Beijing, China; ^2^ Department of Andrology, China-Japan Friendship Hospital, Beijing, China

**Keywords:** diabetes mellitus, erectile dysfunction, bibliometric analysis, visualization, research hotspots

## Abstract

**Background:**

The prevalence of diabetes mellitus-induced erectile dysfunction (DMED) has recently increased, which has prompted numerous DMED studies. Here, we conduct a bibliometric analysis of relevant literature in the field of DMED and to discuss the research hotspots and future development directions.

**Methods:**

The Web of Science Core Collection database was searched for literature on DMED, and literature characterization including the number of articles, journals, countries/regions, institutions, authors, keywords, and other information was performed using VOS viewer and CiteSpace software. In addition, Pajek software was used for visual map adjustment, and GraphPad Prism was used to generate line graphs.

**Results:**

A total of 804 articles concerning DMED were included in this study. *The Journal of Sexual Medicine* issued the most documents(92 articles). The United States and China were in the leading position in the field of DMED research, and cross-institutional collaboration on DMED research worldwide needs to be further strengthened. Ryu JK were the authors with the highest number of documents issued (22 articles) while Bivalacqua TJ was the author with the most co-citated(249 co-citated). The keywords analysis shows that the main research hotspots in the field of DMED were mechanism discussions and disease treatment and management.

**Conclusions:**

Global research on DMED is expected to increase further. The investigation of the mechanism of DMED and the exploration of new therapeutic means and targets are the focus of future research.

## Introduction

Erectile dysfunction (ED) refers to a common male sexual dysfunction disease in which the penis cannot achieve or maintain an adequate erectile status for sexual performance when receiving effective stimulation (1). Currently, more than 150 million people worldwide suffer from ED, and the number of patients is expected to exceed 322 million by 2025 (2). Risk factors for ED include age, obesity, smoking, depression, and metabolic disorders (3), which are more common in middle-aged and elderly men with diabetes (4, 5). Long-term hyperglycemia in diabetic patients induces the production of large amounts of reactive oxygen species that impair mitochondrial and smooth muscle function in the corpus cavernosum, which leads to ED (6, 7). In different regions of the world, the prevalence of diabetes mellitus-induced erectile dysfunction (DMED) ranges from 35.8% to 86.1% (8), and 12% of diabetic patients have ED as the first symptom (9). The incidence of ED is higher in patients with type 1 diabetes because of more pronounced blood glucose fluctuations (10). Studies have shown that the prevalence and severity of DMED are positively correlated with the duration of diabetes (11). Some patients have difficulty speaking about ED early in the disease and do not receive a systematic diagnosis and treatment; therefore, they will progress to moderate to severe ED, which in turn increases the risk of coronary and cerebrovascular diseases (12). In addition, with the change of life rhythms and the improvement of quality of life, DMED has a significant tendency for rejuvenation (13), which not only poses more challenges to the treatment and management of DMED but also seriously affects the quality of life and mental health of patients. To date, many scholars have studied the epidemiology, mechanism, treatment, and prevention of DMED, but the literature is redundant and unsystematic, which is not conducive to the overall understanding of the field by researchers. Therefore, a generalized analysis of the literature related to DMED is needed.

Bibliometric analysis is a method to quantitatively explore and analyze all knowledge carriers in a certain field by using certain statistical methods, which can help us understand the research hotspots, knowledge structure, cooperation mode, and future development direction in a certain field and can provide a certain basis for the formulation of clinical guidelines (14, 15) and has been widely used in various fields. The aim of this paper is to summarize the characteristics of DMED literature, explore the research results and future development trends in this field, and provide references for future research (16).

## Materials and methods

### Procedure

The Web of Science Core Collection (WoSCC) database was searched on August 5, 2022, by subject terms from January 1, 1992 to July 31, 2022, using the search formula “Topic (diabetic OR diabetes OR diabetes mellitus) AND Topic (erectile dysfunction OR impotence).” Original research, review, and meta-analysis articles on DMED were included. Conference abstracts, conference proceedings, case reports, letters, articles unrelated to the topic, unpublished documents without enough information for further analysis, and duplicates were excluded. Articles were exported as a plain text file, and the title, keywords, institution, journal, country or region, authors, and references of each article were collected. As this paper is a bibliometric study, neither human participants nor animals were involved, no ethical approval was necessary.

### Data analysis

VOSviewer 1.6.18 (https://www.vosviewer.com/download) was used for co-occurrence analysis of institutions, authors, countries, and keywords, which includes three modules: network visualization, overlay visualization, and density visualization, and the relationship between scientific literature and research hotspots was mapped multidimensionally in a visual atlas (17). CiteSpace 5.8 R3 (https://citespace.podia.com/) was used for keyword clustering and burst word analysis, the time node was selected from January 1992 to July 2022, the time slice was one, node type was “keywords,” and default values were used for other parameter options. In addition, Pajek software (http://mrvar.fdv.uni-lj.si/pajek/) was used for visual map adjustment, and GraphPad Prism 8.0.2 (http://www.graphpad-prism.cn/) was used to count the number of documents issued and generate line graphs.

## Results

### Annual publications

**Figure 1:**
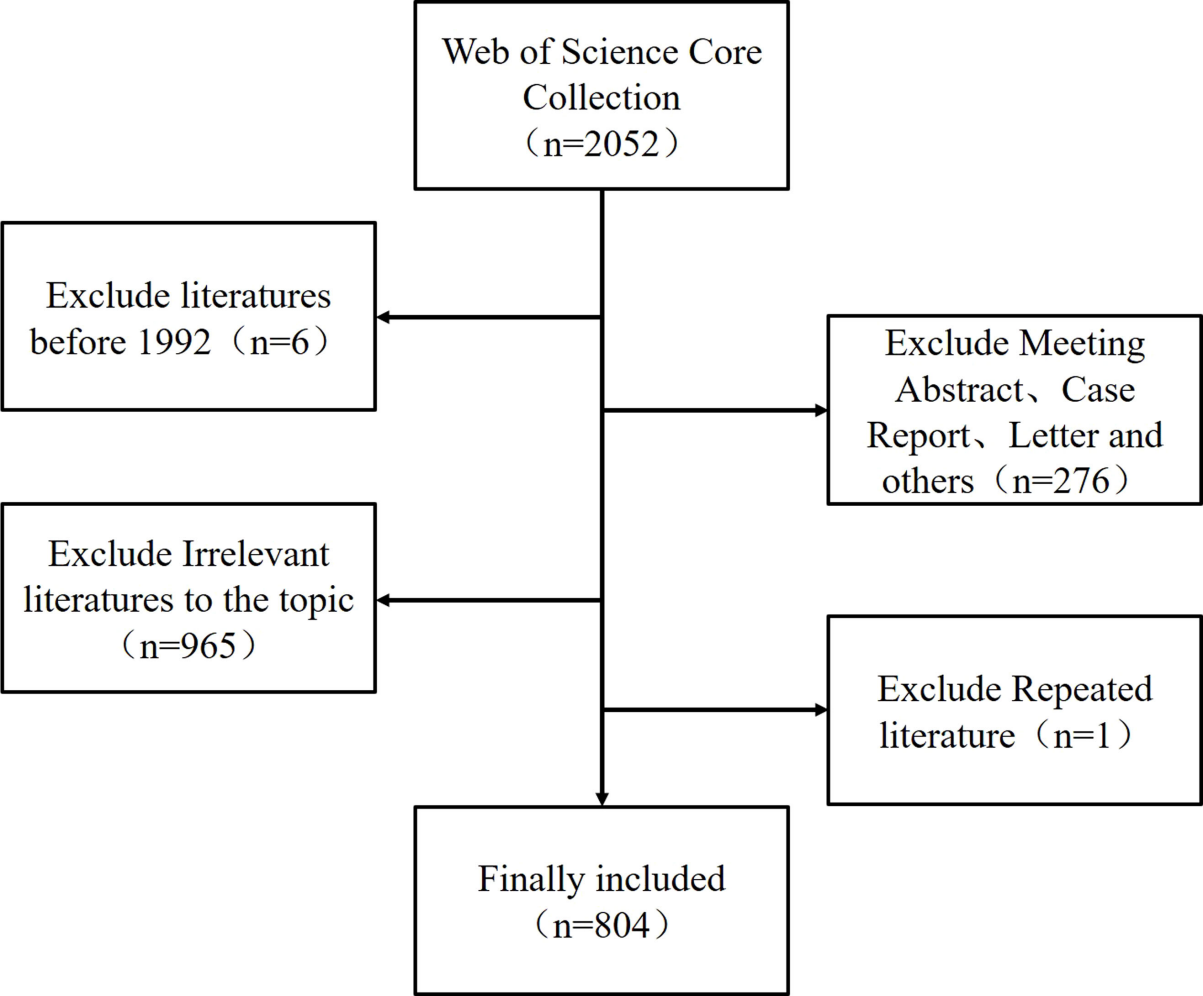
Flow diagram of the included papers.

**Figure 2:**
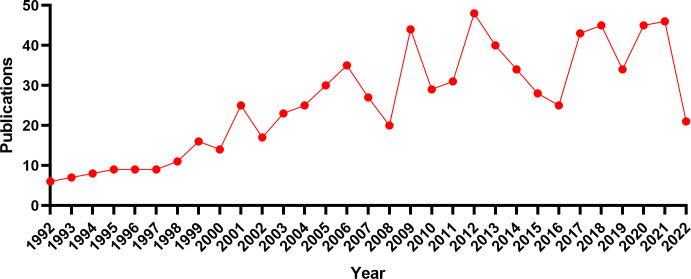
Annual publication volume trend from 1991 to 2022.

A total of 804 articles (**Figure 1**) were included in this study, and a line graph was obtained by applying statistics to the annual number of documents issued (**Figure 2**). The annual number of documents issued for the DMED study showed a fluctuating upward trend, with six peaks in 2001, 2006, 2009, 2012, 2018, and 2021, indicating a higher research intensity in these years. The decrease in the number of documents issued in 2022 was related to the search time point, and the number of documents issued showed an increasing trend over time. In the past 5 years, the annual number of documents issued is at a high level, indicating that DMED has received increasingly more attention and remains a hotspot for current research.

### Analysis of leading journals

A total of 313 academic journals published articles on DMED. **Table 1** shows the top 10 journal names and the corresponding number of documents issued by each one. *Journal of Sexual Medicine* topped the list with 92 articles, followed by the *International Journal of Impotence Research* (n = 49), the *Journal of Urology* (n = 30), and *Andrologia* (n = 29). In addition, the *Journal of Sexual Medicine* (3576 times) was cited the most frequently by the top 10 journals, followed by *Diabetes* (2216 times), the *International Journal of Impotence Research* (1638 times), and the *Journal of Urology* (1599 times), indicating that these journals have strong academic influence in the field of DMED.

**Table 1:** The top 10 productive and cited journals on the research of DMED.

Ranking	Journal	Publications	Count of citations	IF(2022)
1	Journal of Sexual Medicine	92	3576	3.937
2	International Journal of Impotence Research	49	1638	2.408
3	Journal of Urology	30	1599	7.600
4	Andrologia	29	231	2.532
5	Asian Journal of Andrology	22	503	3.054
6	BJU International	21	622	5.969
7	Andrology	15	182	4.456
8	Diabetes Care	14	2216	17.152
9	European Urology	13	508	24.267
9	Urology	13	260	2.633

### Analysis of leading countries, regions, and institutions

**Figure 3:**
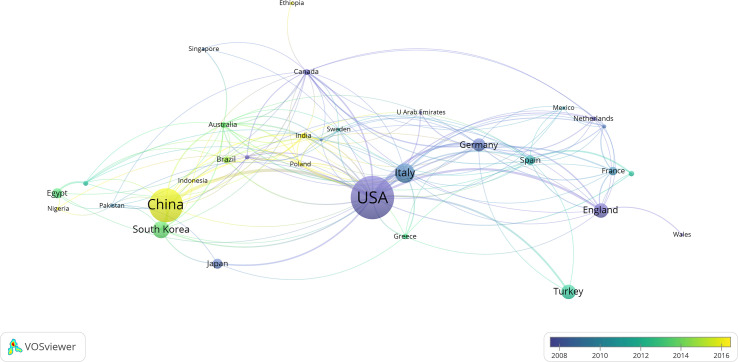
The network map of countries and regions.

The analysis of national and regional cooperative network maps for DMED-related research (**Table 2**; **Figure 3**) showed the 15 countries or regions that contributed the most to DMED research, with the United States publishing 212 articles ranking first, followed closely by China with 152 articles, Italy (n=71), South Korea (n=59), England (n=47), and Turkey (n=47). The size of the circle located in each country represents the number of documents issued, with different colors representing different time intervals, and the thickness of the lines represents the intensity of cooperation. This indicates that different regions of the world have conducted different degrees of research on DMED, and in terms of the intensity of associations, the top five are the United States, China, Germany, Italy, and England, indicating that cross-regional and cross-national research cooperation is also increasing.

**Table 2:** The top 15 productive countries/region on the research of DMED.

Ranking	Countries/Region	Publications	Link Srength
1	USA	212	117
2	China	152	46
3	Italy	71	37
4	South Korea	59	22
5	England	47	30
5	Turkey	47	9
7	Germany	40	41
8	Egypt	28	11
8	Japan	28	9
10	Spain	26	26
11	France	22	18
12	Brazil	18	10
13	Canada	15	32
14	Australia	14	33
15	India	13	19

**Figure 4:**
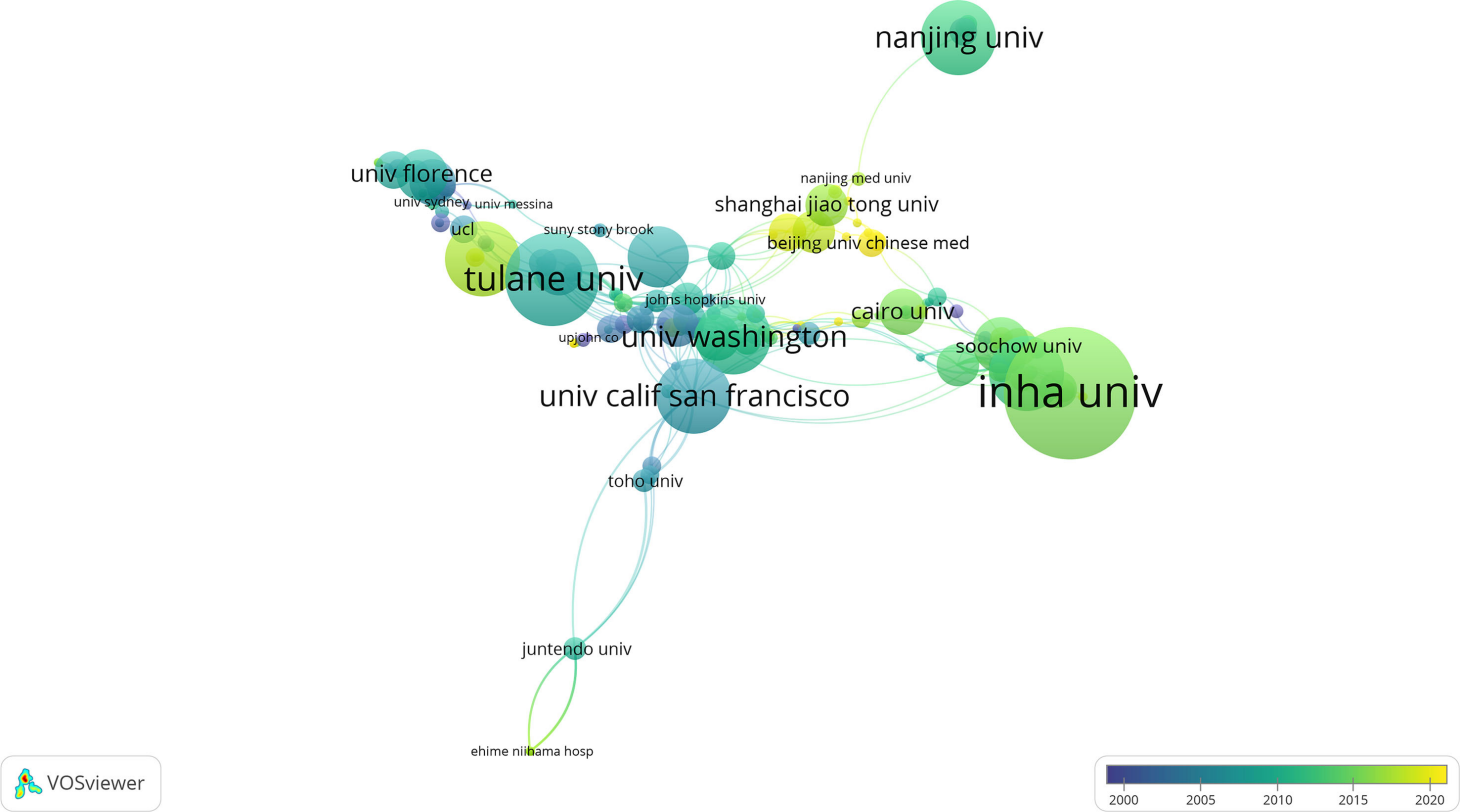
The network map of institutions.

**Table 3** shows that the top three institutions that contributed most to DMED research were Inha University (n=28), Huazhong University of Science and Technology (n=22), and Tulane University (n=20). The institutional network collaboration map (**Figure 4**) and institutional collaboration link strength show that cross-institutional collaboration on DMED research worldwide needs to be further strengthened.

**Table 3:** The top 10 productive institutions on the research of DMED.

Ranking	Institutions	Publications	Link Srength
1	Inha University	28	21
2	Huazhong University of Science and Technology	22	0
3	Tulane University	20	16
4	Ankara University	16	5
4	Nanjing University	16	2
4	Seoul National University	16	11
4	University California San Francisco	16	16
4	University of Washington	16	12
9	Albert Einstein College of Medicine	13	4
10	Peking University	12	11

### Analysis of leading authors and co-cited authors

**Figure 5:**
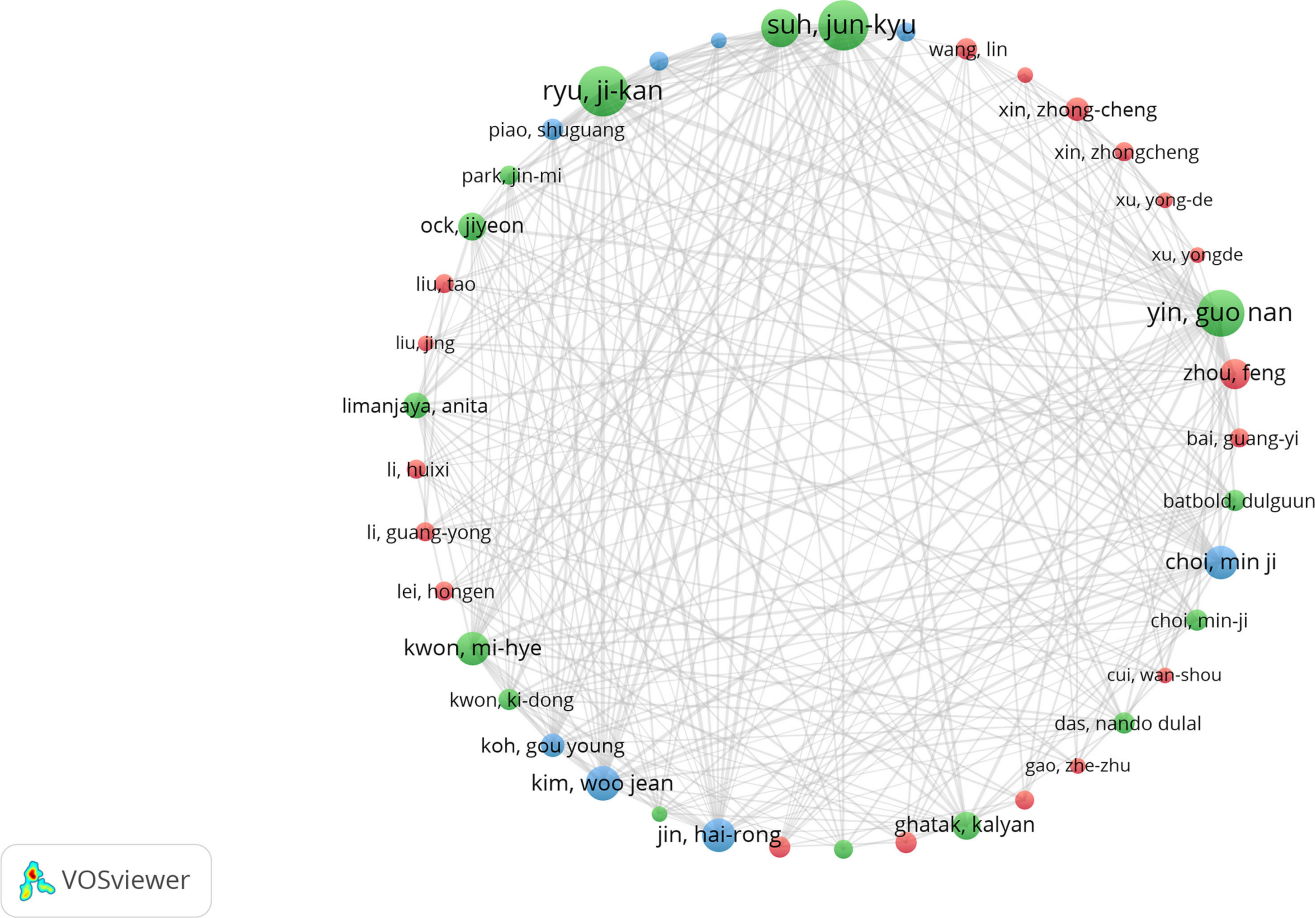
Cooperation map of authors.

Circles of different colors in the co-occurrence map of the author cooperation network (**Figure 5**) represent different clusters, and lines represent their cooperation intensity. This analysis indicates three major author cooperation groups and that extensive cooperation has been carried out among authors. **Table 4** shows Ryu JK (22 articles), Suh JK (22 articles), Yin GN (20 articles), Wang T (16 articles), and Gur S (14 articles) as the top five authors with the most documents issued. The authors with the most co-citated are Biacqua TJ (249 co-citated), Feldman HA (243 co-citated), Corona G (239 co-citated), Rosen RC (213 co-citated), and DeTejada IS (175 co-citated); all of them have high academic influence in the field of DMED.

**Table 4:** The top 10 productive and cited Auhtors on the research of DMED.

Ranking	Author	Documents	Co-cited author	Count
1	Ryu JK	22	Bivalacqua, TJ	249
2	Suh JK	22	Feldman, HA	243
3	Yin GN	20	Corona, G	239
4	Wang T	16	Rosen, RC	213
5	Gur S	14	DeTejada, IS	175
6	Liu JH	14	Esposito, K	159
7	Song KM	14	Goldstein, I	154
8	Kim WJ	12	Burnett, AL	148
9	Chen Y	11	Lue, TF	134
10	Choi MJ	11	Fedele, D	126

### Analysis of keywords and burst words

**Figure 6:**
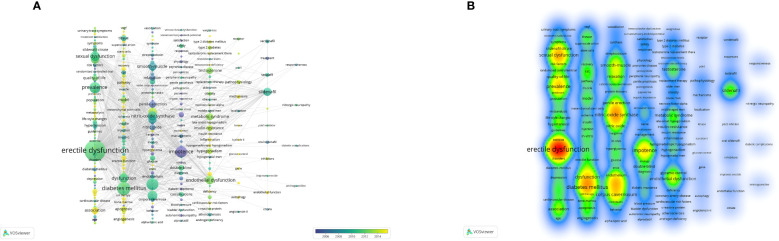
Analysis of keywords. **(A)** co-occurring map of keywords. Note: Each keyword font and the size of the circle represent the frequency of occurrence of the keyword; the thickness of the line represents the strength of association between each keyword; and different colors in the circle represent different time intervals. **(B)** keyword density map. Note: Each keyword font and its annulus brightness represent how often the keyword appears.

Keywords are the generalization and extraction of the central thought of an article, and their frequency of occurrence is positively correlated with the amount of focus on the related research field, and thus, research hotspots and the shifting focus can be grasped by analyzing the keywords (18). In this study, 300 keywords with a frequency of occurrence ≥ 5 were included in the analysis. In addition to erectile dysfunction, impotence, diabetes, and other subject headings, the five keywords with the highest frequency of occurrence were prevalence(112 occurrences), corpus cavernosa (112 occurrences), sexual dysfunction (108 occurrences), nitric-oxide synthase (101 occurrences), and endothelial dysfunction (89 occurrences). The research hotspots covered various fields of epidemiology, basic medicine, and clinical medicine (**Figures 6A, B**).

**Figure 7:**
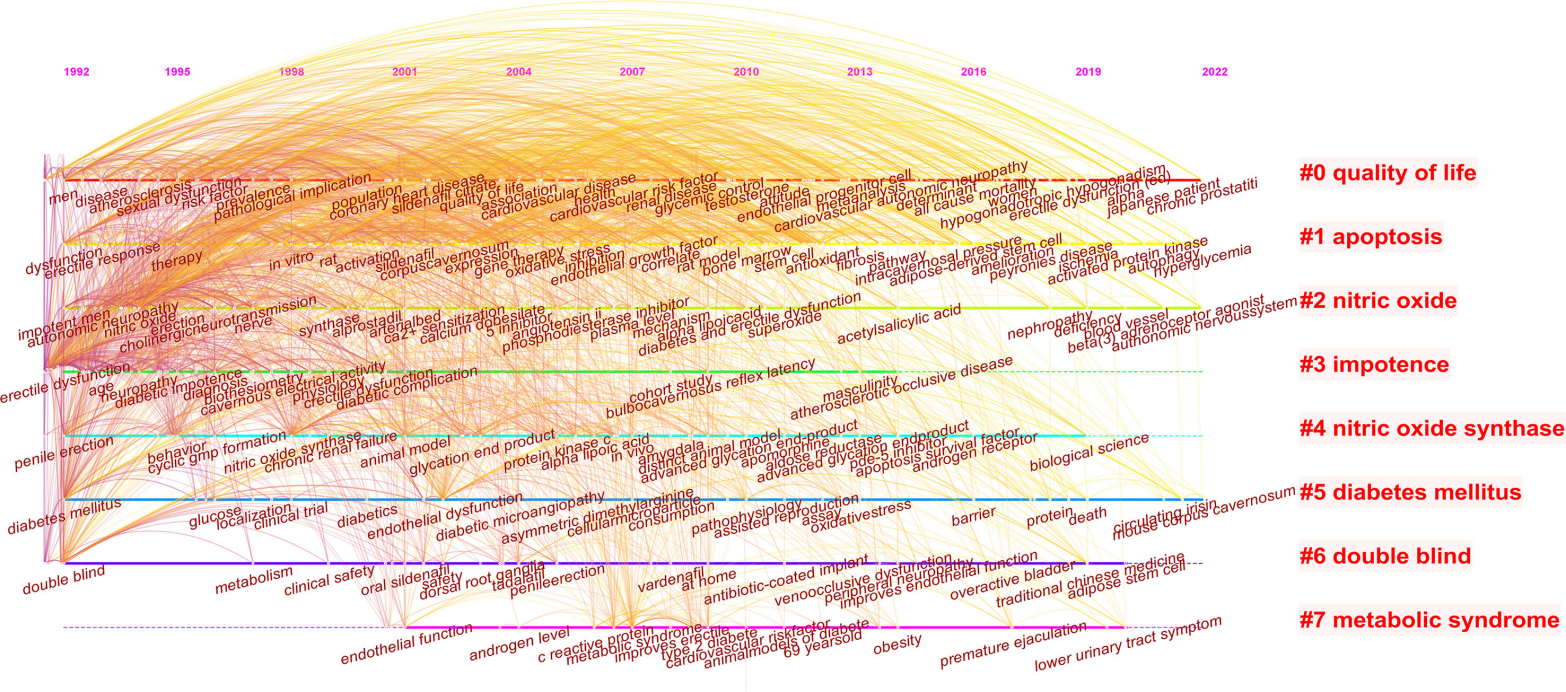
Keyword timeline chart and keyword clusters.

The keywords were clustered to form a timeline map (**Figure 7**), according to the frequency of keyword usage over time, and research progress can be visualized. Eight clusters were formed in this study, which were quality of life, apoptosis, nitric oxide, impotence, nitric oxide synthase, diabetes mellitus, double blind, and metabolic syndrome.

**Figure 8:**
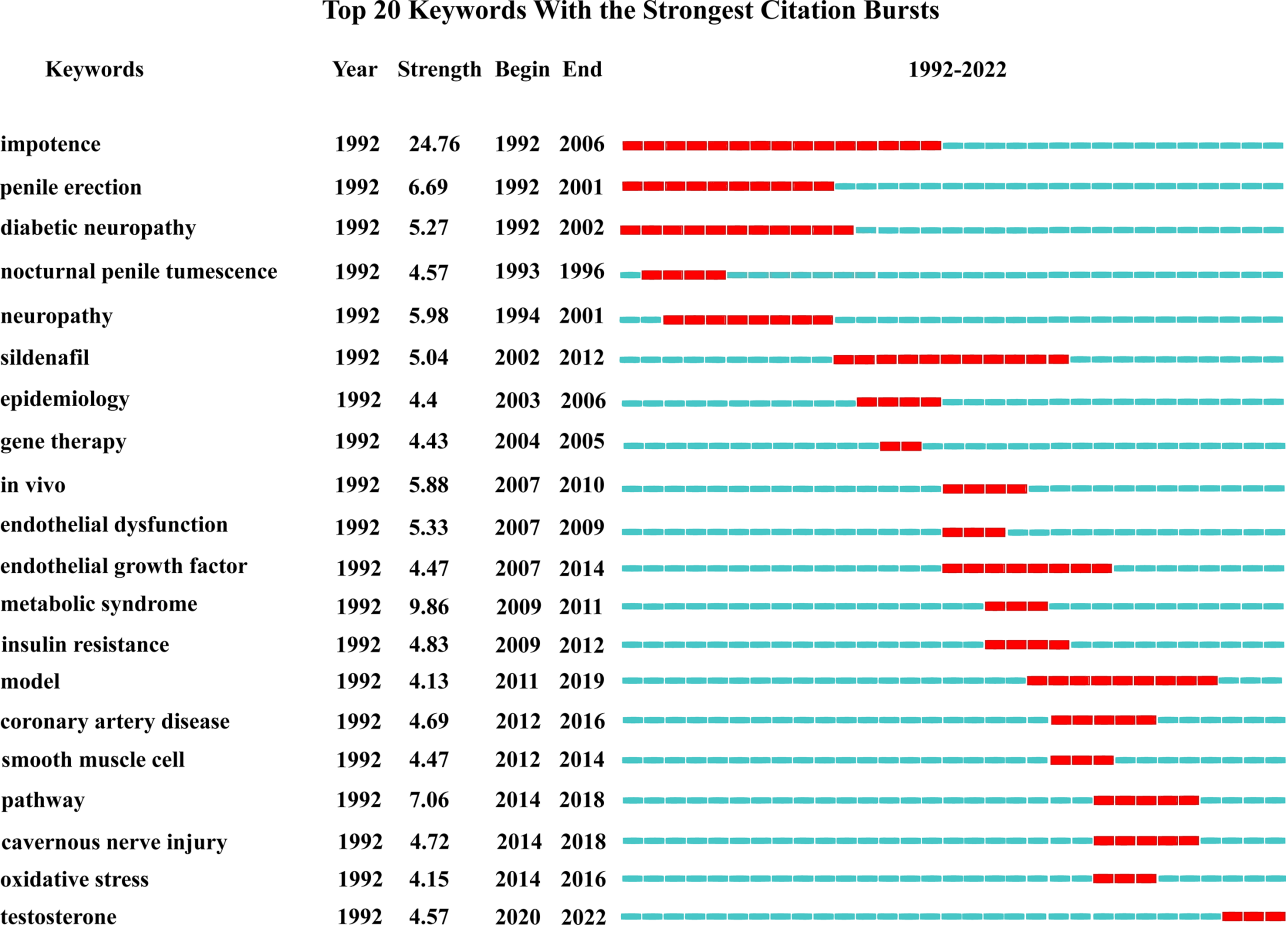
Top 20 keywords with the strongest citation bursts.

If the frequency of occurrence of a keyword increases significantly within a short period of time, it is called a burst word, which can reflect the research hotspots in that period of time, and according to the time of emergence and disappearance of burst words, we can also roughly grasp the research trend and development direction (19). Burst words in this study had undergone a transition from the recognition of impotence and penile erection disease itself to the exploration of the mechanism of DMED (**Figure 8**).

## Discussion

### General information

In recent decades, the prevalence of diabetes mellitus (DM) has continued to rise. From 1980 to 2014, the number of diabetic patients worldwide increased from 108 million to 422 million, nearly 4-fold, becoming the fourth largest non-communicable disease in the world (20). Elevated blood glucose is significantly related to reduced male sexual function, and 55%-80% of DM male patients suffer from ED (21, 22), with a pathogenesis involving endocrine, neurological, psychological, vascular, and other lesions (23). Phosphodiesterase type 5 (PDE5) inhibitors are used as a first-line drug choice for DMED treatment, but the mechanism of its regulatory influence on blood glucose is still unclear, and 40% of patients still have no treatment response (24), which limits its use in treatment. With the increasing prevalence of DMED, higher requirements have been put forward for its diagnosis, treatment, and management. Therefore, the number of studies on DMED has been increasing in recent years, and the annual number of articles published has remained at about 40.

The United States and China are in the leading position in the field of DMED research, and the study of DMED in Asia is becoming more in-depth. The reason for this is related to the epidemiology of DM. The study showed that the countries with the highest number of DM cases worldwide in 2011 were China (90 million), India (61.3 million), and the United States (23.7 million), and the number of DM cases in these three countries is expected to increase to 129.7 million, 101.2 million, and 29.6 million, respectively, by 2030, with 48% of the increase expected to occur in China and India (25). The increase in the incidence of diabetes and its related complications means an increase in national funding and scientific research investment, and it is expected that all countries and regions will strengthen their cooperation to promote further DMED research.

From the perspective of authors and journals, Ryu JK issued the highest number of documents on DMED and formed a collaborative team with authors such as Ryu JK and Yin GN, whose research on DMED mainly focused on the mining and mechanistic exploration of novel therapeutic targets, such as vasohibin−1 and injurin 1 expressed by penile endothelial cells, which are expected to be new targets for DMED treatment to improve penile blood supply by mediating angiogenesis (26, 27). The author who has been co-cited the most frequently in the literature is Bivalacqua TJ, whose research mainly focused on investigating the mechanism of endothelial nitric oxide synthase (eNOS) in DMED (28, 29), which shows that the research hotspots of DMED focus on the mechanism of occurrence and treatment and continue to deepen. The *Journal of Sexual Medicine*, the *International Journal of Impotence Research*, and the *Journal of Urology* are the top three journals in terms of the number of articles published, and they all contain high-quality scientific results, with an average of 36.73 citations per article and an average impact factor of 4.65, indicating that these journals have high influence and authority in the field of DMED.

### Hotspots and frontiers

Combined with the frequency of occurrence of keywords, timeline mapping, and burst word mapping, the research hotspots and development directions in the field of DMED are summarized.

1. Mechanism of DMED: Since the mechanism of DMED is complex and involves vascular, neurological, and endocrine factors, more new proteins or pathways related to its development are being discovered as research continues to progress.

Oxidative stress and endothelial damage: Decreased synthesis or bioavailability of eNOS and in the penile vascular endothelium of DM patients due to oxidative stress damage is essential to the development of ED (30). Studies have shown that hyperglycemia-induced advanced glycation end products lead to the production of large amounts of reactive oxygen species and reactive nitrogen species (31), increased oxidative stress, decreased eNOS synthesis, cavernous endothelial dysfunction, and abnormal smooth muscle diastole, leading to the development of ED (32, 33). Moreover, protein oxidation changes in the corpus cavernosum of DM rats were significantly increased, mainly manifested as nitration of tyrosine residues to form 3-nitrotyrosine, which directly inactivated NO and induced apoptosis in endothelial cells (32). DMED is also associated with upregulation of toll-like receptor (TLR)4 expression, which contributes to oxidative stress and the release of pro-inflammatory cytokines in damaged vessels (34). Enhanced TLR4 expression was observed in corpus cavernosum from diabetic rats compared with control animals and anti-TLR4 antibody improved erectile function in diabetic rats due to attenuation of oxidative stress and increased cGMP levels in penile tissue (35).

Neurovascular injury and repair: As a secreted glycoprotein, Dickkopf3 regulates the Wnt signaling pathway and upregulates angiopoietin-1, vascular endothelial growth factor (VEGF), and basic fibroblast growth factor, exerting angiogenic and protective functions. However, its expression was significantly reduced in the penile corpus cavernosum of DMED mice, impeding cavernous endothelial cell repair and angiogenesis (36). The nerve growth factor precursor protein (proNGF) has a high affinity for the p75 neurotrophin receptor (p75NTR). Hyperglycemia-induced overexpression of p75NTR and disrupted homeostasis of NGF and proNGF levels resulted in a significant upregulation of the proNGF/p75NTR pathway in mouse cavernous epithelial cells, accelerating neuronal cell apoptosis and inducing nerve injury (37).

Endocrine disorders: DM was shown to promote apoptosis in testicular cells by affecting Bcl-2 and caspase protease expression, leading to decreased testosterone levels, structural changes in smooth muscle cell, which also contributed to the development of ED (38, 39).Improving the understanding of the molecular mechanisms underlying the development of DMED could contribute to the development of more effective treatments.

2. Continuously innovative treatment: PDE5 inhibitors are used as the first-line treatment for DMED. Clinical studies have shown that the efficiency of sildenafil in the treatment of DMED is 46.3% with good safety, and patient satisfaction with their sexual life is improved after taking the drug orally (40). Animal experiments have also confirmed that sildenafil improved erectile function by increasing the expression of VEGF and eNOS in the penis of diabetic rats (41). For hypogonadal patients with testosterone deficiency, testosterone replacement therapy may be used (42). On the basis of the above-mentioned traditional treatment, drugs more commonly used in other fields have been introduced in the treatment of DMED. This has realized a new use of older drugs, such as valsartan (43), acetylcysteine (44), and melatonin (45), to improve erectile function by inhibiting the activation of the local renin-angiotensin system (RAS) in the cavernous body and reducing oxidative stress and the inflammatory response, all of which have shown good efficacy. In addition, nutritional supplements composed by *panax ginseng*, *moringa oleifera* and *rutin* have been reported to improve erectile function through extends the activity of the chronic treatment with Tadalafil and enhance the endothelial NO and cGMP production (46). In terms of second-line treatment, intracavernosal injection of angiogenic factors and neurotrophic factors is currently the focus of research (47, 48).

However, although PDE5 inhibitors are the first-line therapeutic agents for DMED, their effects depend on the production of endogenous NO (49). Severe vascular and neurological dysfunction in diabetic patients leads to decreased endogenous NOS activity; so, some patients have an extremely poor response to PDE5 inhibitor therapy (50). The mining of new treatments has become the main direction of future research, including stem cell therapy and gene therapy. Studies have shown that stem cell transplantation upregulated the autophagic activity of cavernous cells, which differentiated to endothelial cells and smooth muscle cells to repair cell damage and then restored erectile function in DM patients (51). Adipose-derived stem cells are more frequently used in DMED treatment due to their more convenient acquisition, and their effects were not affected by blood glucose levels (52). Gene therapy is also a new direction. Target genes such as vascular nerve regeneration factor (53) and insulin-like growth factor 1 (IGF-1) (54) are significantly downregulated in patients with advanced DMED, and repair therapy with these genes also improved erectile function. In addition, low-intensity extra corporeal shock wave therapy (Li-ESWT) for DMED is also a topic of interest in current research, which is a non-invasive therapy with few side effects that can be used as a treatment option for patients with a poor response to oral drugs (55). Li-ESWT improved the local blood supply of the penis and promoted nerve regeneration with an efficiency of 71% in the treatment of DMED (56). If all of the above measures are ineffective, penile prosthesis implantation can also be considered. The three-piece inflatable penile prosthesis (IPP) is the most common implant used in penile surgery with the highest patient satisfaction (57).

3. Comprehensive management of DMED: The incidence of ED in DM patients is three times higher than that in non-DM patients, and age and duration of diabetes are independent risk factors for the occurrence of DMED (58). Elderly diabetic patients also often suffer from obesity, hypertension, hyperlipidemia, and other manifestations of metabolic syndrome, which are considered to be independent risk factors for endothelial dysfunction, ED, and cardiovascular disease (59). Therefore, in clinical practice, the assessment and management of ED should be an important part of the follow-up process of DM patients, and early diagnosis and treatment of DMED are important for controlling risk factors and improving patient quality of life (60). Furthermore, clinicians should take psychological aspects into consideration when treating DMED and should actively attend to the patient’s psychological health and help regulate the patient’s negative emotions (61).

## Conclusion

Over the past three decades, research on DMED has been increasing. Papers published in professional journals have received more attention than those published in comprehensive journals, and the deepening of research can be further promoted by enhancing cooperation between countries, institutions, and authors. DMED is a promising field in which treatment and management are essential to improve the quality of life of patients. According to the current growth trend, it is expected that global research on DMED will be increased further, and the further exploration of its mechanism and the mining of new treatment means and targets are the directions of future research.

## Strengths and limitations

The strength of this study is to analyze the global research status of DMED from a bibliometric perspective, providing valuable information for relevant researchers in terms of authors, journals, institutions, research hotspots, and research frontiers. A limitation of this study is that only the WoSCC database was included for analysis because the academic literature in this database is considered the most valuable and reliable. Meetings, books, and other article types were not included in the data collection process, which is also one of the reasons for the limited number of articles (62).

## Data availability statement

The raw data supporting the conclusions of this article will be made available by the authors, without undue reservation.

## Author contributions

LZ and BB designed the study. JG, ZQ, HH, LC conducted the literature search. LZ, BB analyzed the data, LZ wrote the manuscript. BL supervised the study and revised the manuscript. All authors contributed to the article and approved the submitted version.
